# Emotional development among early school-age children: gender differences in the role of problem behaviours

**DOI:** 10.1080/01443410.2015.1034090

**Published:** 2015-04-23

**Authors:** Lisa K. Maguire, Ulrike Niens, Mark McCann, Paul Connolly

**Affiliations:** ^a^ Centre for Public Health, Queen’s University Belfast, Belfast, UK; ^b^ School of Education, Queen’s University Belfast, Belfast, UK; ^c^ Institute for Child Care Research, Queen’s University Belfast, Belfast, UK

**Keywords:** classroom behaviour, social and emotional learning, gender differences

## Abstract

There has been an increasing focus on social and emotional development in educational programmes in early childhood as both variables are believed to influence behavioural outcomes in the classroom. However, relationships between social and emotional development and behaviour in early childhood have rarely been explored. This article sets out to investigate the conceptualisation of these variables and their interrelationships. Structural equation models were used to assess whether differences exist between boys and girls in relation to social and emotional competences, which could affect the relative success of such programmes. This article is based on cross-sectional data collected from 749 four- to six-year-olds and their teachers. The findings generally supported the hypothesised relationships between social and emotional development variables and prosocial behaviour (including internalising behaviour) for boys and girls. However, some gender differences were noted in externalising behaviour, which teachers often consider to be most significant due to its potentially disruptive nature in the classroom.

## Introduction

The importance of schools focusing on the development of social and emotional skills in pupils is now widely accepted (Denham & Brown, 2010; Gillies, 2011; Greenberg et al., 2003). The recognition that emotions can facilitate prosocial behaviour and learning has developed from the understanding that ‘schools are social places and learning is a social process’ (Zins, Bloodworth, Weissberg, & Walberg, 2007, p. 191). In such social contexts, educators often focus on reducing so-called problematic behaviours, which include internalising behaviours (such as social withdrawal, depression) and externalising behaviours (such as aggression, disruption). Concerns about problematic behaviours relate to their long-term impact on the individual child as well as the potentially disruptive impact of externalising behaviours in particular on the classroom. Longitudinal research indicates that problematic behaviours that develop in early childhood may be stable all the way through into adolescence for some individuals (Campbell, 1995; Moffitt, Caspi, Dickson, Silva, & Stanton, 1996) and where boys have been found to be more effected by such behaviours than girls (Kaiser, Cai, & Hancock, 2002). Children displaying externalising behavioural problems at a young age are at a greater risk of developing antisocial behaviour, juvenile delinquency and other behavioural disorders in the future (Campbell, 2006; Lynam, 1996). As such, it is important to better understand the mechanisms behind such problematic behaviour and its interrelationship with socio-emotional learning (SEL) in order to prevent its initial occurrence and to develop appropriate educational programmes on SEL for the early years. However, the literature surrounding the concepts is at times confused and contradictory and more clarity is needed about the direction of relationships between key variables and potential gender differences. This has been a point highlighted by Wigelsworth, Humphrey, Kalambouka, and Lendrum (2010) who discuss the inconsistent terminology used in the literature to date.

Several large-scale randomised control trials of SEL programmes have been conducted in recent years (e.g. Bywater, Hutchings, Whitaker, Evans, & Parry, 2011; Domitrovich, Cortes, & Greenberg, 2007) and results on the outcomes, specifically relating to externalising behaviours, have been favourable. However, it is not known whether these programmes are best implemented universally within classrooms or tailored to specific social categories, such as gender. Moreover, the theoretical basis of such programmes, in particular the directionality of relationships between key variables, has often not been tested in previous research.

This article presents the findings of a large-scale cross-sectional study investigating the relationships between socio-emotional variables and behaviours and explores whether these relationships are moderated by other key factors, in this case gender. Findings from structural equation models support the use of universal school-based programmes but also present some evidence that challenges the existing theoretical basis of such interventions. In particular, the article demonstrates that gender differences do exist in relation to how socio-emotional variables and externalising problematic behaviours relate to one another, and this is used to suggest that some tailoring of programmes may be beneficial in order to take into account differences between groups of children. While the focus of this article has been gender, it is suggested that these findings may also be true for other key variables such as age, race/ethnicity and socio-economic status, and thus further research in this area is required.

In the following, we will consider relevant key concepts in turn, namely social behaviour, SEL and inhibitory control, and discuss their theoretically proposed relationships with reference to the empirical evidence.

## Problematic behaviour, SEL and inhibitory control

### Problematic behaviours

Problematic behaviours are highly prevalent in early childhood (Bagner, Rodríguez, Blake, Linares, & Carter, 2012) and pose a risk for children of developing problems in later life. While some researchers believe such early problems to be transitory (Briggs-Gowan, Carter, Bosson-Heenan, Guyer, & Horwitz, 2006), Lavigne et al. (1998) have found that 50% of children displaying externalising behaviour and diagnosed with a disruptive behaviour disorder at ages two to three continue to have this diagnosis two years later which demonstrates that, for some, these problems do appear to be stable over time. Children with high levels of externalising behaviours have been found to have many adjustment difficulties and are at risk of developing social and academic problems (Doumen, Verschueren, & Buyse, 2009), which early intervention may successfully alleviate (e.g. Murray, 2010; Shaw, Dishion, Supplee, Gardner, & Arnds, 2006). Research indicates that boys tend to experience greater difficulty than girls in controlling aggression and higher levels of externalising disorders (Steinberg, 2008). Indeed, there is a body of research highlighting gender differences in aggressive behaviours in early childhood (Baillargeon et al., 2007).

Classroom behaviour is an important part of school life, and managing behaviour effectively impacts on learning, interpersonal relationships and the emotional health of both teachers and pupils. Externalising behaviour can be most damaging for teaching and learning as it is able to ‘upset the pace of work and obstruct coexistence’ (Esturgó-Deu & Sala-Roca, 2010). Classroom behaviour can also have an impact on educational attainment. Research indicates that prosocial behaviours tend to predict academic success and that poor educational attainment occurs in conjunction with externalising behaviour (e.g. Malecki & Elliot, 2002; Masten et al., 1995). Research has also found that both prosocial and problematic behaviours are influenced by social and emotional processes (Osher, Bear, Sprague, & Doyle, 2010) and that young children displaying problematic behaviours also tend to have deficits in social and emotional skills (Bierman & Welsh, 1997). Caprara, Barbaranelli, Pastorelli, Bandura, and Zimbardo (2000) highlight the importance of prosocial behaviours in the classroom which they found to predict academic achievement five years later even after previous achievement was held constant. In a cross-sectional study of four-year-old children, Bierman, Torres, Domitrovich, Welsh, and Gest (2009) found similar relationships between behaviour and academic knowledge whereby socio-emotional skills, measured through teacher assessments, were highlighted as important predictors and the prediction was particularly strong for girls. The authors concluded that gender variations relating to academic knowledge were due to differences in behaviour rather than cognitive skills and that further research using multiple measurement methods was required to investigate such gender differences in the early years.

### Social and emotional learning

Social and emotional learning has been defined as

the process through which children acquire the knowledge, attitudes and skills to recognise and manage their emotions, set and achieve positive goals, demonstrate caring and concern for others, establish and maintain positive relationships, make responsible decisions and handle interpersonal situations effectively. (Payton et al., 2008, pp. 5–6)

Social and emotional learning thus involves a range of factors including identifying, interpreting and expressing emotions, which are clearly defined to be closely linked to social behaviours. However, the terminology used to discuss these in the literature seems inconsistent and has been described as ‘notoriously slippery and ill-defined’ (Gillies, 2011). As such, this study attempted to focus on these variables by clearly differentiating between concepts and identifying their role in determining prosocial and problematic behaviour.

Emotional understanding, which incorporates the ability to recognise and appraise emotions, is a fundamental social task (Halberstadt, 2003). Emotional recognition depends on the ability to identify facial expressions that contain a significant amount of emotional information. The ability to discriminate this information emerges in the first year of life (Flom & Bahrick, 2007) and continues to develop into adulthood (Thomas, De Bellis, Graham, & LaBar, 2007). Research indicates that some emotions are more easily discriminated than others: scared and happy faces were recognised easier than neutral faces, whereas sad faces had the lowest accuracy and longest response times (Tottenham, Hare, & Casey, 2011). In general, girls were also found to be more accurate than boys at discriminating between emotions (Tottenham et al., 2011). Leerkes, Paradise, O’Brien, Calkins, and Lange (2008) found that emotional understanding was related to academic achievement in pre-schoolers and there is a growing body of evidence which suggests that problematic behaviour is predicted by a lack of skills relating to emotional recognition and appraisal (Dodge, Laird, Lochman, & Zelli, 2002). For example, studies show that children displaying externalising behaviours may not recognise emotions in others accurately and tend to attribute anger to others particularly in ambiguous situations (Schultz, Izard, & Ackerman, 2000).

As children develop through primary school, they are increasingly able to identify emotions (Selman, 1981) and to interpret them within specific social contexts. This enables them to express their own emotions competently in their social environment. Children’s developing skills in emotional understanding, including recognition and appraisal – alongside the development of competent emotional expression – permit them to navigate the complex social and academic school environment successfully and to develop prosocial behaviours. The classroom can be a confusing and disorientating place for children who are unable to accurately identify emotions or interpret the impact of specific contexts on other people’s emotions (Raver, Garner, & Smith-Donald, 2007), and this may result in children displaying externalising behaviours that disrupt classroom learning. It is therefore important to further our understanding of the interrelationship between key variables involved in emotional understanding and competent emotional expression and their impact on behaviour.

Esturgó-Deu and Sala-Roca (2010) note that there have been few studies that focused on the link between emotional understanding and social behaviours. Their research indicated that a lack of emotional abilities was related to a reduced ability to self-regulate emotions and increased externalising behaviour. Inhibitory control has been suggested as an essential element of self-regulation, which in turn may impact on social behaviour (Homack & Riccio, 2004). Yücel et al. (2012) define inhibitory control as a ‘broad term referring to the ability to flexibly adapt behaviour’. Components include delayed gratification, inhibiting impulsive behaviours and the capability to plan and organise activities (Yücel et al., 2012). Emotional, cognitive and behavioural inhibitory control processes are believed to be reciprocally interconnected (Blair & Razza, 2007; Pessoa, Kastner, & Ungerleider, 2002). Tottenham et al. (2011) found evidence that emotional information can impair cognitive controls. Processing emotional information can affect the ability to provide – and may actually suppress – appropriate responses to social contexts (Verona, Patrick, & Lang, 2002). The role of regulating emotion in the context of social interactions is important as it is believed that inhibiting impulsive or dominant responses to conflict may help to prevent children from reacting aggressively in challenging situations (Orobio de Castro, Bosch, Veerman, & Koops, 2003).

Emotional and inhibitory control are believed to be reciprocally interconnected (Blair & Razza, 2007; Pessoa et al., 2002). While Tottenham et al. (2011) found evidence that emotional information can impair cognitive controls, a study by Verona et al. (2002) indicated that processing emotional information can affect the ability to provide, and may actually suppress, appropriate responses. Indeed, neurobiological models of children’s school readiness have suggested relationships between inhibitory control and both socio-emotional and academic functioning (Blair, 2002) and are therefore seen to be an important area to target for intervention (Riggs, Greenberg, Kusché, & Pentz, 2006). Despite such interventions generally being implemented universally for both boys and girls, research highlights the role of gender and emotions in inhibitory control. Matthews, Ponitz, and Morrison (2009) found that girls performed better than boys in behavioural regulation. Other researchers have found a difference in reaction times as an indicator of inhibitory control on such tasks and found that, in comparison with females, males were consistently slower on some whilst being faster on others. This is explained by the fact that the different inhibitory control tasks require the use of different processing areas of the brain which are known to be more dominant for men or women (Mekarski, Cutmore, & Suboski, 1996). Chang, Olson, Sameroff, and Sexton (2011) found that a lack in regulatory skills, such as inhibitory control, predicted increased externalising behaviours in later life for boys but not girls. The authors propose ‘a sex-differentiated pathway to externalising behaviour across the transition from preschool to school entry’ (Chang et al., 2011, p. 79). Yücel et al. (2012) note that impairments of inhibitory control affect many behavioural, personal and social problems, and it has been frequently highlighted that components of SEL – such as emotional understanding, including recognition and appraisal, competence in expressing emotions as well as inhibitory control – do not exist in isolation but depend on each other and are likely to be multidirectional (Denham & Brown, 2010; Miller et al., 2006). Researchers have put forward differing theories on how these concepts are associated. Denham and Brown (2010) propose that future research should pay particular attention to the mechanisms and processes which link components of SEL and focus on identifying groups who may vary in SEL characteristics as developing specific interventions for these groups would be ‘more strategic’ (p. 665).

Many educational programmes focussing on prosocial and problematic behaviour in early childhood today comprise elements of SEL. Such programmes are usually implemented on a ‘universal’ basis, in that they are delivered to all pupils within a school context. A recent meta-analysis synthesising the findings of 213 studies indicates that these programmes are generally effective with regard to behavioural and other outcomes (Durlak, Weissberg, Dymnicki, Taylor, & Schellinger, 2011). The authors suggest that future research needs to be theory driven in order to accurately assess skills and identify how they are interrelated. There have been a number of classroom-based universal prevention interventions reported in the literature which have found a differential effect of the intervention on gender (Garner, Mahatmya, Brown, & Vesely, 2014). The promoting alternative thinking strategies programme (PATHS), for example, have found stronger effects for boys (Bierman et al., 2010). Moreover, long-term follow-up of a universal intervention to reduce aggressive, anti-social and disruptive behaviour found that whilst beneficial effects were found for all, boys responded particularly well (Kellam et al., 2008). Bradshaw, Zmuda, Kellam, and Ialongo (2009) describe these differential effects as potentially be due to underachieving girls receiving less attention from teachers than their male counterparts and calls for in-depth research on these differences to help to modify future universal programmes to enhance their success. In their review, Garner et al. (2014) also found that gender differences become more salient when paired with ethnicity and socio-economic status. Durlak et al. (2011) also recommend that future research should focus on subgroup analysis to determine whether factors such as gender may be related to differential benefits.

### The present study

In the light of the above discussion, this article examines the psychometric and structural characteristics underpinning these emotional development constructs using contemporary data analytic methods. The constructs are then tested to determine their relationships with aspects of children’s behaviour and to examine potential gender differences with regard to these relationships.

Due to the complex and overlapping nature of the concepts discussed, clear evidence of their importance and relationship with other variables is not readily discernible from the literature. Many studies looking at these concepts are based on relatively small datasets which were analysed with respect to outcomes of specific educational initiatives. There has been little clarity in the theoretical literature about the direction that relationships between concepts are assumed to take and whether the relationships between these concepts operate in the same way for boys and girls, despite the well-documented gender differences relating to SEL and behaviour noted above. A better understanding of the role of these concepts and their relationships may thus be crucial to enable educators to develop and differentiate SEL programmes effectively and appropriately.

### Research aims

The aims of this study were to investigate the theoretical concepts of emotional understanding, emotional competence and inhibitory control and their impact on classroom behaviour and to identify whether gender differences exist in how these concepts affect classroom behaviour. The research hypotheses are as follows:

(1) higher levels of emotional expression, emotional understanding and inhibitory control are positively related to prosocial behaviour and negatively related to internalising and externalising classroom behaviour;
(2) the effect of emotional understanding on classroom behaviours is mediated through emotional expression and inhibitory control;
(3) there are gender differences in emotional expression, emotional understanding, inhibitory control and classroom behaviour, with girls showing higher levels of inhibitory control, emotional understanding and expression, more prosocial and internalising behaviour and less externalising behaviours than boys;
(4) these gender differences are reflected in the associations between emotional constructs and classroom behaviours.

## Method

### Participants

The study took place in 12 primary schools in Northern Ireland utilising a cross-sectional correlation design. Ethical approval for the study was sought and obtained from the Research Ethics Committee, at the authors’ institution. Recruitment and data collection were carried out between September 2008 and January 2009. Data were collected in the context of an evaluation for an SEL programme that was implemented in the classroom after baseline measurement. The data reported here relate to the initial baseline data sweep, before the SEL programme was implemented.

Children in the Year 1 and Year 2 class groups were identified as the groups of interest for this study as these are the first two years of formal education in Northern Ireland. Parents of children in participating schools received a letter to explain the research and were given the opportunity to opt-out of the research. Also, the nature and purpose of the research was explained to each child prior to their participation, and they also had the opportunity to opt-out of the research. In total, 749 children took part: 356 girls (47.5%) and 393 boys (52.5%). A total of 380 participants (50.7%) were in their first year of schooling (who would normally be between four and five years old) and 369 (49.3%) were in Year 2 (who would normally be between five and six years old). As the consent process was managed by the primary schools, accurate opt-out rates could not be gathered. The participants came from 38 different classes located in twelve schools.

A researcher visited each classroom to test the children on an individual basis. The researcher read aloud each question and recorded the child’s answers on the questionnaire. The benefit of testing in this fashion was that the researcher was able to explain any complicated words or phrases used and check that the children understood what each question meant before responding. The researchers (all trained teachers) belonged to a pool of researchers commonly used to collect data in research conducted within our institution and therefore were experienced in data collection methods with young children. They received a half day of training on the use and administration of the specific research instruments used in this study.

### Measures

*Emotional understanding* was measured using two instruments – the *Assessment of Children’s Emotional Skills* and the *Emotional Recognition Questionnaire*. The *Assessment of Children’s Emotional Skills* (ACES; Schultz, Izard, & Bear, 2004) questionnaire assessed children’s emotional recognition skills. Children were presented with 12 photographs of children of a similar age who are demonstrating facial expressions. The facial expressions used were happy, sad, angry and scared. Children were asked to identify the emotion the child in the picture was experiencing from a choice of happy, sad, angry, scared, or no feeling. Results were coded and summed to create an emotion attribution accuracy score reflected how many photographs a child answered correctly (range 0–12). Reliability was found to be acceptable with a Cronbach’s Alpha of .992. The *Emotion Recognition Questionnaire* (Ribordy, Camras, Stefani, & Spaccarelli, 1988) consists of 16 vignettes, with four vignettes for each of four emotions (happy, sad, angry, and scared) which assessed emotional appraisal. Children were shown four pictures of a character experiencing each of the four emotions. After ensuring that the children could identify the emotion depicted in each of four pictures, the researcher would read each vignette (the gender in each vignette was matched to the gender of the child) and the child would choose the emotion that they felt the child in the story was experiencing by selecting a picture of one of the four facial expressions. Example vignettes were as follows: ‘Claire was the only one in class not to get any Valentines cards on Valentine’s Day. Did Claire feel happy, sad, mad or scared?’ and ‘Jack had an older brother who was telling Jack ghost stories. Did Jack feel happy, sad, mad or scared?’. Responses were coded as either correct or incorrect, and the total score ranged from 0 to 16. Cronbach’s Alpha for this measure was found to be .591. The ERQ was developed and used with children in grades 1 to 6 (Bierman et al., 2008; Conduct Problems Prevention Research Group, 1999). The ACES was initially developed with a group of 182 children aged between 6 and 9 and has been used in evaluations of the PATHS programme with children as young as 3 years old (Domitrovich et al., 2007).

*Inhibitory control* was measured using the tapping test by Luria (1966). The tapping test measures the ability to suppress imitation and inhibit interfering responses. The test requires that the child taps twice in response to a single tap from the examiner, or once in response to two taps (maximum score = 10). Cronbach’s Alpha was found to be acceptable for this measure at .860.

#### Competent emotional expression

Teachers completed a brief measure of emotional competence that was derived from the PATHS evaluation kit (Greenberg & Kusche, 2005) and consisted of twelve items measured by a six-item Likert scale ranging from ‘Never’ to ‘Almost always’. Total possible scores ranged from 0 to 60 with higher scores, indicating greater levels of emotional competence. Examples of questions included are ‘Recognises and label his/her feelings accurately’ and ‘Stops and calms down when frustrated or upset’. The measure had high reliability with Chronbach’s Alpha = .961.

#### Classroom behaviour

The teacher version strengths and difficulties questionnaire (SDQ; Goodman, 1997) was used as a measure of classroom behaviour. Class teachers were asked to complete the questionnaire. The SDQ contains 25 items and measures psychological attributes across five scales: emotional symptoms, conduct problems, prosocial behaviour, hyperactivity and peer problems. Items are scored on a scale ranging from 0 to 2 (not true, somewhat true and certainly true) with total scale scores ranging between 0 and 10. Based on previous theoretical and empirical research (Goodman, Lamping, & Ploubidis, 2010), three scales were used as follows: prosocial behaviour, internalising behaviours and externalising behaviours. The Internalising scale contained the emotional symptoms and peer problems subscale and the Externalising scale was comprised of the conduct problems and hyperactivity subscales and could be seen as most closely related to aggressive and disruptive behaviour in the classroom. Psychometric properties of the SDQ have been found to be satisfactory (Goodman, 2001) and ranged between .771 and .902 across the different subscales in this study.

### Data analytic strategy

The data were entered into SPSS Version 17.0©. SPSS was used for descriptive analyses, and MPlus Version 7.11© was utilised for structural equation modelling (SEM). Three-level SEM was used to account for the clustering of participants within classrooms within schools. These models did not suggest that accounting for between-school variation led to different interpretation of the models; hence, the models presented below are based on single level models with robust standard errors to account for clustering. Based on the literature, two theoretical models were tested. The first suggests that emotional understanding influences both inhibitory control and competent emotional expression, and inhibitory control also influences competent emotional expression. Emotional competence alone has a direct influence on classroom behaviour, while the influence of emotional understanding and inhibitory control are mediated through competent emotional expression. The second model suggests that emotional understanding also has a direct influence on classroom behaviour independent of competent emotional expression. The influence of inhibitory control on classroom behaviour is solely mediated through its influence on competent emotional expression. The model contains three latent variables: emotional understanding (determined by observed scores on the emotion attribution accuracy and the emotion recognition questionnaire scales); internalising behaviour (determined by SDQ emotional difficulties and peer problems scales); and externalising behaviour (determined by SDQ hyperactivity and conduct problems scales). Inhibitory control, competent emotional expression and SDQ prosocial behaviour scales were modelled as observed variables.

Descriptive statistics were obtained for observed variables, and *t*-tests used to assess gender differences in their distribution. Correlations between all variables were calculated, and SEM was used to assess gender differences for associations between variables estimated from the best fitting model. Global fit was assessed using the comparative fit index (CFI), the Tucker Lewis index (TLI), and the root mean square approximation error (RMSEA). Acceptable fit indices were taken as CFI and TLI values as above .90 (Kline, 2004) and values of RMSEA below .05 (Byrne, 1998). Multiple group analysis examined whether the structural paths between variables differed for boys and girls. Equality constraints were imposed on each parameter sequentially to test for invariance of the path between boys and girls. The Satorra-Bentler scaled chi-squared difference test was used to assess the difference between the null model (with parameter constraints) and the alternative model (without constraints). These tests indicated whether structural paths differed between boys and girls.

## Results

### Descriptive statistics and correlations

Table 1 contains a summary of the study variables for the whole sample. The skewness and kurtosis values for the study variables met the criteria for normality. There were gender differences in levels of emotional regulation, emotional competence, emotional recognition, hyperactivity and prosocial behaviour. Table 2 contains the correlations for all the variables. Competent emotional expression was positively correlated with the emotional understanding measures and the prosocial scale of the SDQ, and negatively correlated with the emotional, conduct, hyperactivity and peer problems scales. This was true for both the whole sample and the separate gender groups, although the correlation between competent emotional expression and the emotional problems scale of the SDQ was stronger among girls.

**Table 1.** Descriptive statistics for all study variables.

Variable	*M* (SD)	Kurtosis	Skewness	Range
Whole sample				
*Emotional understanding*				
Emotional attribution accuracy (Accuracy)	5 (1.64)	2.5	−.27	0–8
Emotion recognition questionnaire (ERQ)	10.21 (2.63)	2.68	−.29	2–16

*Inhibitory control*				
Emotional regulation (EReg)	87.49 (18.98)	8.34	−2.26	0–100

*Competent emotional expression*				
Emotional competence (EComp)	36.28 (11.9)	2.55	−.11	1–60

*Classroom behaviour*				
Emotional symptoms (EES)	1.13 (1.73)	5.89	1.78	0–9
Conduct problems (CPS)	.76 (1.42)	10.40	2.56	0–10
Hyperactivity (HS)	3.34 (2.98)	2.49	.76	0–10
Peer problems (PPS)	1.36 (1.65)	4.65	1.35	0–9
Prosocial behaviour (PS)	6.93 (2.35)	2.51	−.45	0–10

**Table 2.** Intercorrelations of the study variables.

	EReg	ERQ	Accuracy	ProSoc	EES	CPS	HS	PPS	EComp
Whole sample									
EReg	–								
ERQ	.32**	–							
Accuracy	.23**	.25**	–						
ProSoc	.34**	.28**	.14**	–					
EES	−.08*	−.18**	−.05	−.13**	–				
CPS	−.22**	−.09*	−.06	−.47**	.16**	–			
HS	−.34**	−.21**	−.12**	−.55**	.11**	.57**	–		
PPS	−.25**	−.23**	−.14**	−.48**	.32**	.37**	.38**	–	
EComp	.34**	.28**	.15**	.74**	−.24**	−.54**	−.61**	−.54**	–

*Boys*									
EReg	–								
ERQ	.34**	–							
Accuracy	.21**	.21**	–						
ProSoc	.31**	.23**	.14**	–					
EES	−.07	−.12*	−.03	−.18**	–				
CPS	−.19**	−.02	−.02	−.51**	.23**	–			
HS	−.25**	−.16**	−.07	−.51**	.10	.52**	–		
PPS	−.24**	−.25**	−.13*	−.47**	.32**	.38**	.38**	–	
EComp	.32**	.23**	.11*	.72**	−.27**	−.56**	−.57**	−.52**	–

*Girls*									
EReg	–								
ERQ	.30**	–							
Accuracy	.25**	.29**	–						
ProSoc	.34**	.24**	.15**	–					
EES	−.10	−.25**	−.07	−.09	–				
CPS	−.26**	−.14**	−.09	−.42**	.09	–			
HS	−.44**	−.25**	−.19**	−.53**	.14**	.65**	–		
PPS	−.26**	−.23**	−.16**	−.53**	.33**	.38**	.42**	–	
EComp	.35**	.31**	.19**	.72**	−.18**	−.52**	−.62**	−.58**	–

^**^ Correlation is significant at the .01 level (2-tailed).

^*^ Correlation is significant at the .05 level (2-tailed).

### Gender differences in analytical constructs

Table 3 shows the mean scores for boy and girls separately. There were gender differences for several of the constructs under study. No difference appeared for emotional attribution accuracy (*t*_(739) _= .29, *p* = .77), while girls showed better emotional recognition (*t*_(734) _= 2.30, *p* = .02), emotional regulation (*t*_(723) _= 2.10, *p* = .04) and competent emotional expression (*t*_(651) _= 3.93, *p* < .001). In terms of problem behaviours, there were no gender differences in emotional distress (*t*_(740) _= .35, *p* = .07) or peer problems (*t*_(741) _= .17, *p* = .17). There was very weak evidence for a gender difference in conduct problems (*t*_(741) _= −1.66, *p* = .10), tending towards more conduct problems among boys. Finally, there was a much stronger tendency towards greater levels of hyperactivity among boys (*t*_(741) _= −6.11, *p* < .001) and more prosocial behaviour among girls (*t*_(741) _= 5.51, *p* < .001).

**Table 3.** *T*-test and effect size estimates for gender differences in study variables.

Variable	*M* (SD)		Gender difference *t*-test			Effect size Cohen’s *d*
	Girls	Boys	*t*	df	*p* value	
*Emotional understanding*						
Emotional attribution accuracy (Accuracy)	5.01 (1.60)	4.98 (1.67)	.29	739	.77	.02
Emotion recognition questionnaire (ERQ)	10.44 (2.68)	9.99 (2.58)	2.30	734	.02	.17

*Inhibitory control*						
Emotional regulation (EReg)	89.02 (17.16)	86.07 (20.45)	2.10	723	.04	.16

*Competent emotional expression*						
Emotional competence (EComp)	38.17 (11.56)	34.54 (11.96)	3.93	651	<.001	.31

*Classroom behaviour*						
Emotional symptoms (EES)	1.19 (1.74)	1.07 (1.71)	.93	740	.35	.07
Conduct problems (CPS)	.67 (1.44)	.84 (1.39)	−1.66	741	.10	−.12
Hyperactivity (HS)	2.65 (2.77)	3.96 (3.04)	−6.11	741	<.001	−.45
Peer problems (PPS)	1.44 (1.71)	1.28 (1.59)	1.38	741	.17	.10
Prosocial behaviour (PS)	7.42 (2.31)	6.49 (2.31)	5.51	741	<.001	.41

### Structural equation modelling for the entire sample

**Figure 1.**
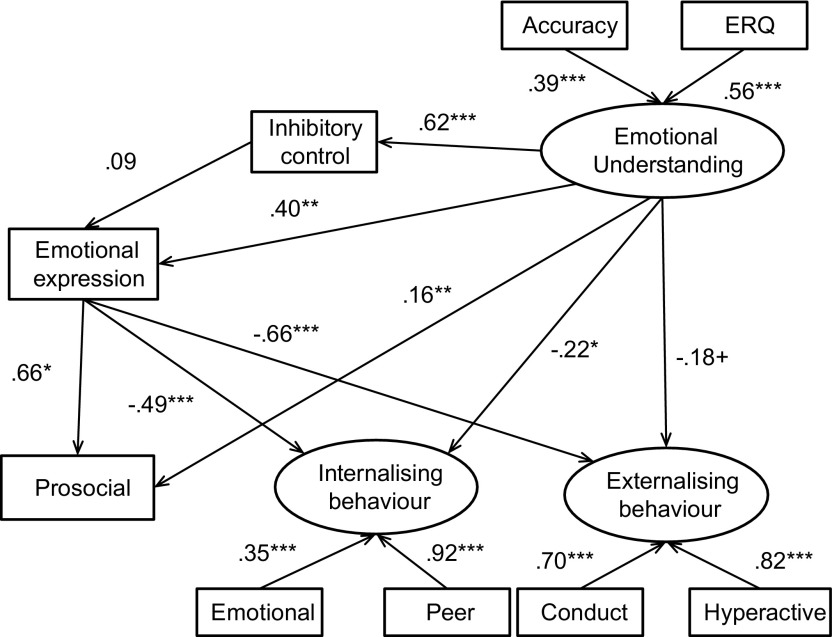
Structural equation model depicting associations between emotional processing and behavioural constructs for the full sample.

Note: Standardised coefficients presented. +*p* < .1; *p < .05; **p < .01; ***p < .001.

Results for the best fitting model are depicted in Figure 1. The final chi-square for this model was *χ* ^2^(18) = 46.07, *p* < .001. CFI and TFI indicated a good model fit (.97 & .94 respectively), and the RMSEA also indicated a good fit (.045) – see Table 4); thus suggesting, overall, that the model was appropriate for the data. The alternative model with no paths linking emotional understanding and behaviour did not provide as good a fit to the data; the sample size adjusted Bayes’ information criterion was 31,817 for the final model, and 31,835 for the alternative model. The fit statistics were adequate, but did not fit the data as well as the accepted final model (RMSEA .49; CFI .96, TLI .93). The final model explained 56% of the variance in prosocial behaviour, 38% of internalising behaviours and 58% of externalising behaviours. Variation in competent emotional expression and emotional understanding was related to the behavioural outcomes: a positive association with prosocial behaviour and an inverse association with internalising and externalising symptoms. This suggests that children with higher levels of emotional understanding and competent emotional expression had lower levels of internalising and externalising behaviours, and higher levels of prosocial behaviour. The indirect effect of emotional understanding (via its effect on competent emotional expression) on all three behavioural outcomes was significant. Around 64% of the effect of emotional understanding on prosocial behaviour was mediated through competent emotional expression (57%) and through emotional expression via inhibitory control (8%). Around 50% of the effect of emotional understanding on internalising behaviour was mediated through competent emotional expression. Around 63% of the effect of emotional understanding was mediated through competent emotional expression.

**Table 4.** Fit statistics for the whole and gender models.

	Whole model	Gender model
		
*Chi-square test of model fit*		
Value	46.07	87.68
Degrees of freedom	18	42
*p*-value	<.001	<.001
		

*RMSEA*		
Estimate	.04	.05
90% C.I.	.03–.06	.04–.07
Prob ≤ .05	.65	.32
CFI	.97	.96
TLI	.94	.93

### Multiple groups structural models for boys and girls

**Figure 2.**
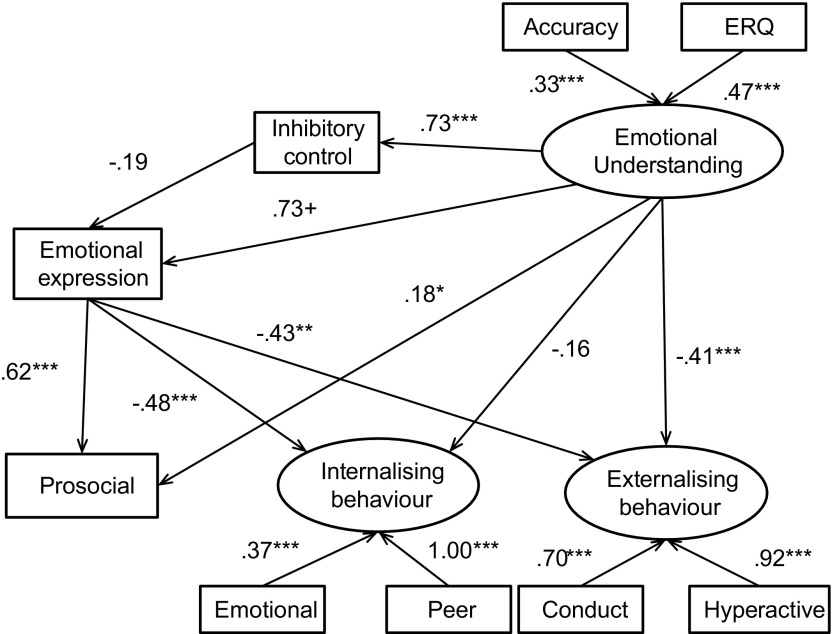
Structural equation model depicting associations between emotional processing and behavioural constructs for the male sample.

Note: Standardised coefficients. +*p* < .1; *p < .05; **p < .01; ***p < .001.

**Figure 3.**
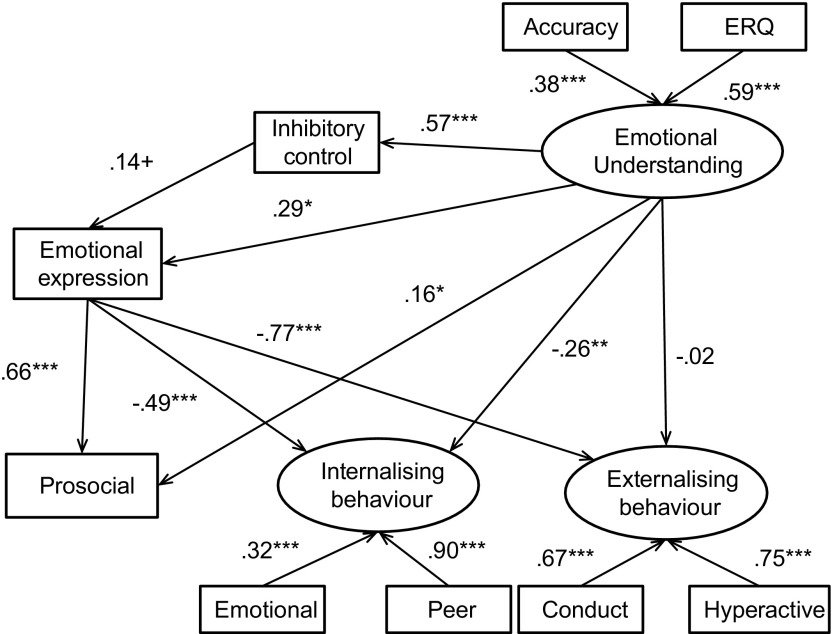
Structural equation model depicting associations between emotional processing and behavioural constructs for the female sample.

Note: Standardised coefficients. +*p* < .1; *p < .05; **p < .01; ***p < .001.

The specified model was then used to predict behavioural outcomes simultaneously for both genders. For boys, the structural equation model (Figure 2) accounted for 54% of the variance in prosocial behaviour, 40% of the variance in internalising behaviours and 61% of the variance in externalising behaviours. For girls, the model (Figure 3) accounted for 55% of the variance in prosocial behaviour, 35% of the variance in internalising behaviours and 56% of the variance in externalising behaviours. The fit for this model was acceptable (see Table 4).

The magnitude and direction of most path estimates were similar to that of the whole sample analysis for boys and girls separately. However, there were some gender differences. The relationship between emotional understanding and internalising behaviour in girls was no longer significant at the .05 level, although there was no evidence that the path varied by gender (Satorra Bentler test .28, *p* = .60). A negative association between emotional understanding and externalising behaviour appeared among girls (*β* = −.41, *p* = .02). However, no such association appeared among boys (*β* = −.02, *p* = .81). The association between competent emotional expression and externalising behaviour also varied between boys (*β* = −.77, *p* < .001) and girls (*β* = −.43, *p* = .002). Compared to a model where these path estimates were constrained to be equal between boys and girls, the model with a different path between emotional understanding and externalising behaviour showed a better fit (Satorra Bentler test 9.35, 1 df; *p* = .002), as did the model for a gender difference between competent emotional expression and externalising behaviour (Satorra Bentler test 7.87, 1 df; *p* = .005).

## Discussion

The general aim of this research has been to illustrate the nature and direction of the relationships between emotional development and social behaviour and the potential impact of gender. This study is original in that it has helped to shed light on the conceptualisation, interrelationships and differentiation of theoretical variables proposed to underpin children’s classroom behaviour and incorporated in educational interventions aimed at improving SEL.

To return to our research hypotheses, we found that competent emotional expression plays an important role as a mediator between the theoretical concepts of emotional understanding and the behavioural outcomes of prosocial, internalising and externalising behaviour. Once emotional expression was accounted for, inhibitory control appeared less important in terms of child outcomes. There was evidence of gender differences in levels of externalising behaviours, inhibitory control and emotional expression and also differences in the relationship between some emotional constructs and child behaviour. While universal programmes aimed at promoting SEL in children may be equally effective for boys and girls, the findings from this study indicate that emotional learning may influence internalising and externalising behaviours differently for boys and girls. While the model uncovered in this study overall clearly held up for boys and girls, some differences were indicated in relation to predictors of externalising behaviour, which has also been described as disruptive classroom behaviour (Parsonson, 2012). Previous research also showed that from an early age, boys are more likely to display externalising behaviours than girls (Moffitt, Caspi, Rutter, & Silva, 2001), which in turn has been related to academic achievement in later childhood (Chen, 2010). Our findings indicated that emotional understanding played a *less important* role in predicting externalising behaviour for boys than girls. On the other hand, competent emotional expression played a *greater role* in predicting externalising behaviour for boys than girls. The gender difference in the role of emotional understanding was of greater magnitude than that for emotional expression. In fact, the former showed no association with externalising behaviour among males. There appear to be several potential explanations for these differences based on gender beliefs and norms within society (Nikapota, 2009). Firstly, social norms surrounding gender differences might mean that emotional understanding is seen as less important to learn for boys than for girls. As such, boys and girls may be encouraged differently to avoid externalising, aggressive behaviour whereby there may be more of an emphasis for girls to identify and understand feelings than for boys. Secondly, in our study, pupil measurements were used for emotional understanding and inhibitory control, but competent emotional expression and behaviour were measured using teacher-rated scales. Gender differences might therefore be a result of teachers attributing boys’ externalising behaviour to their emotional incompetence while they may not find this a likely explanation for girls. Thirdly, it may be the case that among girls the primary determinant of externalising behaviour is emotional understanding, whereas the levels of externalising behaviours among boys are driven by other factors.

Whatever the underlying explanation for gender differences in our model, the results here suggest that improving emotional understanding may help reduce the levels of externalising behaviours among girls, although the same does not appear to be the case for boys.

The results found here have clear implications for classroom management and universal prevention programmes. While SEL still has an important part to play in promoting prosocial behaviours, it may be the case that further approaches are required to improve the rates of externalising problems in the classroom for both boys and girls. Universal programmes may work effectively to promote prosocial behaviours and to reduce internalising behaviours. However, in order to effectively reduce externalising behaviour, which is one of the main concerns for teachers in classrooms today, more differentiated approaches may be required. Durlak et al.’s (2011) article was among the first to suggest that future study into SEL programmes must be theory driven in order to assess how these skills interrelate. This present study has sought to add to the theory base by examining interrelationships and considering the differential effect of gender. The findings will also be of interest to researchers in the area of child development, especially those in the field of emotional intelligence and emotional competence, as the research attempts to clarify the relationships and theory between different aspects of competence.

Some limitations are evident in this study. Firstly, it should be noted that these cross-sectional associations cannot demonstrate the direction of relationships, but they do offer some evidence that further investigation of the effectiveness of SEL for reducing externalising problems is warranted. Secondly, teacher ratings of behaviour and competent emotional expression were used which may not accurately represent the associations with these variables. The SDQ measure of behaviour does have good interrater reliability, but future research should consider whether to use additional informants for the measurement of both these concepts. Unfortunately, we did not have access to any data on the socio-economic status or other demographics such as ethnicity for the participants or the gender or experience of their teachers which is a limitation of this research. One final limitation is the measurements that were used in this study. The ERQ scale, in particular, had quite a low level of reliability (*α* = .591). Whilst this does not invalidate the research described here, it may mean that path differences found are too conservative and we may be missing an even larger effect due to this measure.

On the basis of our findings, we argue that further research is required to confirm gender differences relating to the role of emotional understanding and emotional competence using longitudinal designs, different measurements and samples. This would go some way to differentiate theoretical concepts and their interrelationships as uncovered in our model that may help educational practitioners to identify specific emotional difficulties in pupils. Establishing empirically how emotional understanding, inhibitory control and competent emotional expression predict children’s classroom behaviour may thus enable our theoretical understanding of the conceptual underpinnings of educational interventions and represent one further step in the continuous search for potential improvement of SEL interventions in practice. Through a focus on gender, we have raised the more fundamental point that universal programmes might not be optimal for all children and programmes that have some flexibility built in so that they can deal with differences may be worth considering further. While we have demonstrated this in relation to gender in the present study, it would also be worth acknowledging that such findings may also be applicable to other key variables such as socio-economic status, age and race/ethnicity. Durlak et al. (2011) also acknowledged this by suggesting that future research examine other subgroups such as ethnicity, development level and socio-economic status to determine whether these are related to differential programme benefits. Just as this article has found the potential for differing benefits based on gender, examining these other subgroups would be of interest and should be a priority for those organisations and researchers who are developing preventive programmes. Preventive programmes can and do have a large financial cost and as such they should be targeted at those most in need and whom they will benefit most.

## Conclusions

These findings suggest that the relationship between emotional development and problematic externalising behaviours is robust but may not apply equally across students regardless of gender. This has important implications for programmes that aim to improve externalising behaviour and suggests that universal interventions may need some flexibility to accommodate gender differences in emotional development and social behaviour. The findings from the current study provide a means of further understanding the pathways through which behaviour might be improved and highlight the importance of promoting emotional development in an educational context to positively impact upon problematic classroom behaviour. It would be wrong to conclude from our study that separate SEL interventions should be developed for boys and girls. Considerable variations exist within each group and other factors may be equally, if not more, significant that also need to be taken into account. Rather, our key point is that we have shown how the theoretical basis behind SEL interventions may not apply equally to all children but may vary in relation to wider factors. Thus, we need further research to understand better the nature of these variations, not only in relation to gender but also in relation to other groups. Moreover, it would also be important for evaluations of SEL programmes to focus more on this through exploratory analysis; thereby examining in greater detail than may have been done so far the differential effects of existing programmes.
